# X-ray Cherenkov-luminescence tomography reconstruction with a three-component deep learning algorithm: Swin transformer, convolutional neural network, and locality module

**DOI:** 10.1117/1.JBO.28.2.026004

**Published:** 2023-02-16

**Authors:** Jinchao Feng, Hu Zhang, Mengfan Geng, Hanliang Chen, Kebin Jia, Zhonghua Sun, Zhe Li, Xu Cao, Brian W. Pogue

**Affiliations:** a Beijing University of Technology, Beijing Key Laboratory of Computational Intelligence and Intelligent System, Faculty of Information Technology, Beijing, China; b Beijing Laboratory of Advanced Information Networks, Beijing, China; c Xidian University, Engineering Research Center of Molecular and Neuro Imaging of the Ministry of Education and School of Life Science and Technology, Xi’an, China; d University of Wisconsin-Madison, Department of Medical Physics, Madison, Wisconsin, United States

**Keywords:** x-ray Cherenkov-luminescence tomography, Cherenkov imaging, image reconstruction, Swin-transformer, deep learning

## Abstract

**Significance:**

X-ray Cherenkov–luminescence tomography (XCLT) produces fast emission data from megavoltage (MV) x-ray scanning, in which the excitation location of molecules within tissue is reconstructed. However standard filtered backprojection (FBP) algorithms for XCLT sinogram reconstruction can suffer from insufficient data due to dose limitations, so there are limits in the reconstruction quality with some artifacts. We report a deep learning algorithm for XCLT with high image quality and improved quantitative accuracy.

**Aim:**

To directly reconstruct the distribution of emission quantum yield for x-ray Cherenkov-luminescence tomography, we proposed a three-component deep learning algorithm that includes a Swin transformer, convolution neural network, and locality module model.

**Approach:**

A data-to-image model x-ray Cherenkov-luminescence tomography is developed based on a Swin transformer, which is used to extract pixel-level prior information from the sinogram domain. Meanwhile, a convolutional neural network structure is deployed to transform the extracted pixel information from the sinogram domain to the image domain. Finally, a locality module is designed between the encoder and decoder connection structures for delivering features. Its performance was validated with simulation, physical phantom, and *in vivo* experiments.

**Results:**

This approach can better deal with the limits to data than conventional FBP methods. The method was validated with numerical and physical phantom experiments, with results showing that it improved the reconstruction performance mean square error (>94.1%), peak signal-to-noise ratio (>41.7%), and Pearson correlation (>19%) compared with the FBP algorithm. The Swin-CNN also achieved a 32.1% improvement in PSNR over the deep learning method AUTOMAP.

**Conclusions:**

This study shows that the three-component deep learning algorithm provides an effective reconstruction method for x-ray Cherenkov-luminescence tomography.

## 1 Introduction

X-ray Cherenkov-luminescence tomography (XCLT) is a new tomographic imaging technology that provides a tool for monitoring the biological characteristics of tumor *in vivo* with very high energy megavoltage (MV) x-rays as the excitation source.^1^^,^^2^ It uses a clinical linear accelerator (LINAC) to generate the MV x-rays, which produce Cherenkov light as they pass through biological tissues, and this Cherenkov light becomes an internal excitation optical source that excites molecular phosphors or fluorophores for tomographic imaging. Perhaps even more importantly, when treatment occurs with a dynamic modulated treatment plan with full rotational delivery of the x-rays, there can be a near complete tomographic dataset produced from the multiple angles, producing sinogram like data. However, unlike x-ray tomography, the detectable fluorescence generated in biological tissues for XCLT experiences multiple scattering as it leaves, so it is highly diffused. This results in limited unique measurement sets. As a result, the XCLT reconstruction approach is a severely illposed problem. Because XCLT uses x-ray beams generated by a LINAC to scan the imaging objects, we can increase the number of scanning sheets or optimize the scanning mode to minimize the illposedness and improve the quality of the reconstructed images. In therapeutic use, the scan sequence would be limited to the goal of the therapeutic delivery, but it is common to have hundreds of beam angles in a modern intensity modulated radiotherapy or volumetric modulated arc therapy treatment plan. When used as a pure diagnostic, it is still limited by the delivered dose that would be tolerable to the subject being imaged. These limits to x-ray flux result in limits to the useable data for reconstruction.

Recently, a novel rotational XCLT was proposed; it used a multi-leaf collimator (MLC) to shape high-energy x-ray beam into a thin vertical sheet and scanned the imaging object by translating and rotating the sheet at different positions.^3^^–^^7^ Meanwhile, it used a single-pixel detector to acquire fluorescence or luminescence signals. This scanning method is similar to parallel beam computed tomography (CT); therefore, a filtered back projection (FBP) algorithm was used for image reconstruction.^6^ However, its performance largely depended upon the accuracy of collected sinogram data, and as in all CT reconstructions, more projections always yield a better reconstruction. To reduce the radiation dose from x-rays for XCLT, an incomplete dataset for the sinogram would be acquired with a limited number of projections,^6^ which leads to significant artifacts in reconstructed images.^8^^,^^9^ Therefore, creative introduction of reconstruction algorithms that can improve the image quality with limited projection datasets, as is well developed in the CT reconstruction literature, is needed.

Inspired by deep learning, the data-driven supervised learning methods have attracted great attention in medical image reconstruction.^10^^–^^13^ In general, there are two kinds: image-to-image models^10^^,^^11^ and data-to-image models.^12^^,^^13^ For the image-to-image model, it takes low-quality images reconstructed by traditional algorithms as network input and outputs high-quality images. In this case, the deep learning model is identified as a denoiser. The advantages of the image-to-image model are its fast-training fitting time and easy deployment. However, an image-to-image model directly operates on an image to suppress artifacts, and its performance critically depends on the quality of the input image. Information that is lost during traditional algorithms’ reconstruction cannot easily be recovered. For a data-to-image model approach, such as AUTOMAP,^12^ it takes acquired raw signals as the network input and outputs high-quality images through training a deep neural network, which has more information than an image-to-image model approach. However, it requires a large number of training datasets. In general, both of the models use the convolutional neural network (CNN)-based deep learning architecture to learn hierarchies of structured images and sinogram representations.^14^^,^^15^

Due to the use of a thin sheet in the rotational XCLT, the sampling is extended along the depth direction inside the imaging object, such that each measurement contains all of the signals disturbed along the sheet.^6^ Specifically, each line of a sinogram is sequentially sampled with overlapping information of surrounding sinograms. In other words, one-dimensional components of sinograms heavily correlate with each other. The global characteristic of a sinogram image makes it difficult to be captured with traditional CNNs due to the limited size of the convolution receptive field, which reduces the quality of the reconstructed image. In contrast, transformer-based approaches introduce an attention mechanism to handle the sequential inference tasks in natural language processing^16^ and achieve state-of-the-art performance,^17^^–^^19^ especially in several recent medical imaging tests.^20^^–^^24^ However, self-attention has a quadratic complexity to it. To overcome this, the Swin transformer was developed;^25^ it uses the shifted window attention to model cross-window relationships.^26^ The Swin transformer has the advantage of long-range dependency modeling capability with the shifted window scheme.^25^^,^^26^ Therefore, it can extract the high-quality global information, which is effective for improving the reconstruction performance of XCLT. Thus in this study, a transformer-based deep learning method was applied to this optical tomography, which has a limited data problem.

Considering that the value of each pixel in the sinogram is the sum of the fluorescent signal along the x-ray sheet direction and that the information about fluorescence emission yield is recorded in an intermediate representation, we propose a data-to-image model based on a Swin transformer to directly reconstruct the distribution of emission quantum yield, named Swin-CNN. It has three parts: (1) the basic structure of the Swin transformer is used to extract pixel-level prior information from the sinogram domain; (2) a CNN structure is deployed to transform the extracted pixel information from the sinogram domain to the image domain; (3) a locality module is designed between the encoder and decoder connection structures for delivering features. Its performance was validated with simulation, physical phantom, and *in vivo* experiments.

The remainder of the paper is organized as follows. The forward model and the proposed Swin-CNN reconstruction algorithm are introduced in Sec. 2. In Sec. 3, numerical simulation, physical phantom, and *in vivo* experiments are performed to validate its performance. Section 4 presents the discussion and conclusions.

## 2 Methods

### 2.1 Forward Model

XCLT can be mathematically modeled with a set of coupled continuous wave-domain diffusion equations, which are expressed as follows:^27^^–^^30^

$$\nabla D_{x}(\vec{r})\nabla \Phi_{x}(\vec{r})-\mu_{ax}(\vec{r})\Phi_{x}(\vec{r})=-S(\vec{r}),$$ (1)

$$\nabla D_{m}(\vec{r})\nabla \Phi_{m}(\vec{r})-\mu_{am}(\vec{r})\Phi_{m}(\vec{r})=-\Phi_{x}(\vec{r})\eta \mu_{af}(\vec{r}).$$ (2)

Equation (1) is the Cherenkov excitation field, and Eq. (2) is the fluorescence emission field. Subscripts x and m denote the excitation and emission wavelengths, respectively. $\Phi_{x}(\vec{r})$ is the excitation field at position $\vec{r}$. $\Phi_{m}(\vec{r})$ is the emission field at position $\vec{r}$. $\mu_{ax}(\vec{r})$ and $\mu_{am}(\vec{r})$ are the absorption coefficients, and $D_{x}(\vec{r})$ and $D_{m}(\vec{r})$ are the diffusion coefficients. $\mu_{af}(\vec{r})$ is the fluorophore absorption at the excitation wavelength, and η is the fluorophore quantum efficiency. $\eta \mu_{af}(\vec{r})$ is fluorescence quantum yield. $S(\vec{r})$ is Cherenkov light, which is an internal excitation source induced by a sheet-shaped LINAC beam.

Modeling the thin parallel sheet scan and rotating gantry of the LINAC for different angles, sinogram projections can be obtained by accumulating all of the optical signal intensity generated by each sheet beam along each angle. The forward sinogram projection can be calculated by the Radon transform as

$$p(\theta )=\int_{L}\Phi_{m}(\overset{\to }{r})dl,$$ (3)

where $\Phi_{m}(\vec{r})$ is the intensity of fluorescence emission and p(θ) is the measured sinogram projection data. Discretizing Eq. (3), the XCLT projection model is reformulated in matrix form as

$$P=W\mu_{\eta },$$ (4)

where $\mu_{\eta }$ represents the vectorized fluorescence quantum yield image to be reconstructed, P is the vectorized sinogram projection, and W is the forward projection operator and represents the discrete Radon transform.

### 2.2 Filtered Backprojection (FBP) Algorithm

The aim of the XCLT reconstruction is to recover the distribution of quantum field ${\hat{\mu }}_{\eta }$ from the measured sinogram P. When the FBP algorithm is adapted, ${\hat{\mu }}_{\eta }$ has the form of

$${\hat{\mu }}_{\eta }=W^{T}CP,$$ (5)

where C denotes the discrete filter. However, the FBP tends to generate images with artifacts and intensity imbalance.

### 2.3 Swin-CNN Algorithm

**Fig. 1:**
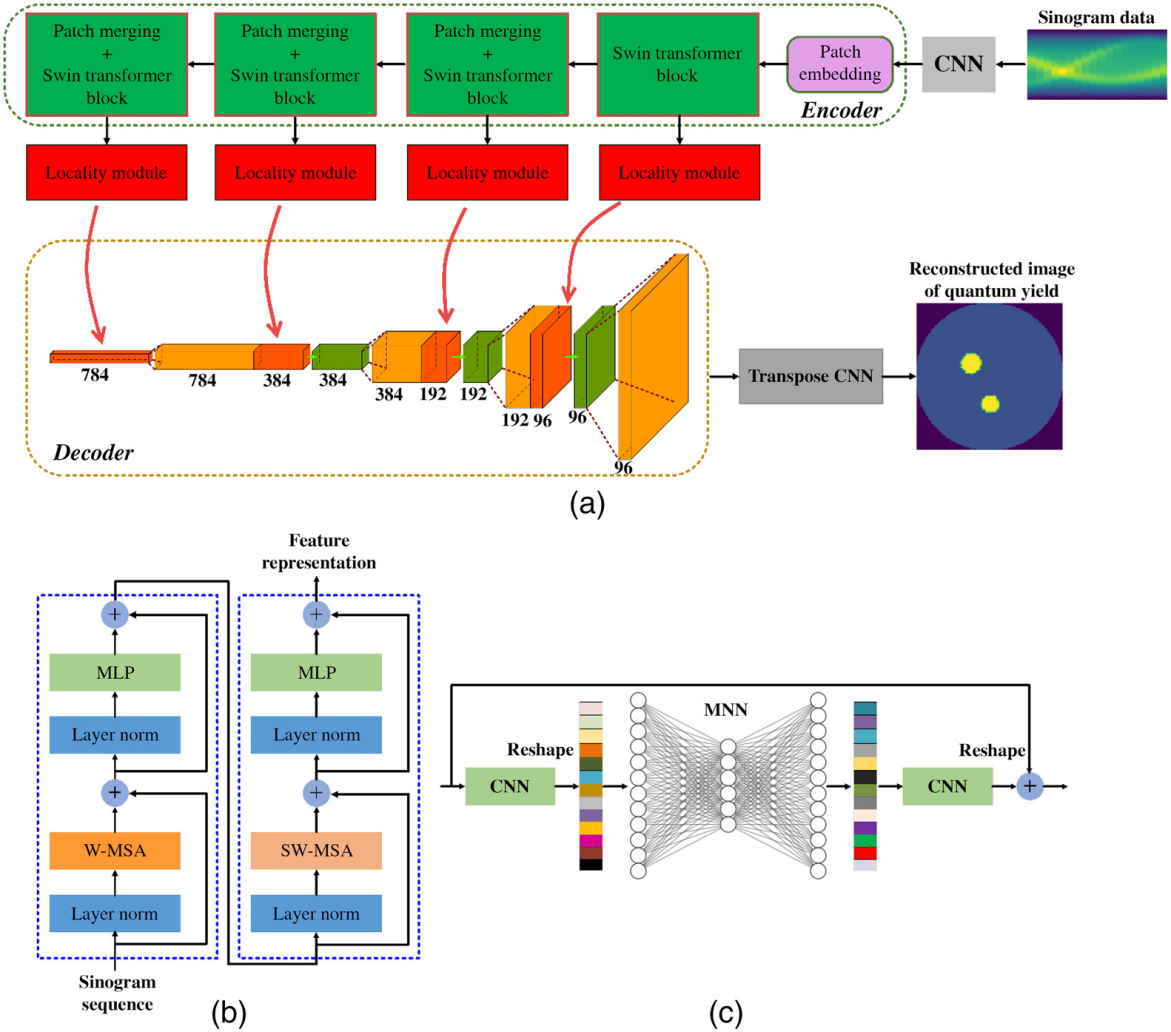
Architectures of (a) the Swin-CNN, (b) the Swin transformer block, and (c) the locality module.

To improve the quality of the reconstructed XCLT image, we develop a deep learning technique to reconstruct fluorescent image through training sonogram projection. It is modeled as follows:

$$f:P\to {\hat{\mu }}_{\eta },$$ (6)

where f is a model with an encode–decode structure, which is shown in Fig. 1(a).

#### 2.3.1 Encoder: Swin transformer

To extract the sinogram pixel feature, a self-attention mechanism is introduced into the encoder based on the Swin transformer block, as shown in Fig. 1(b).

The encoder takes the sinogram image as input and encodes the pixel information into high-level feature representations. More specifically, the sinogram P is first resized into the feature space with the size of N×N×1(N=128), and then a 1×1 basic convolutional layer is applied on feature map for the channel dimension expanding with the size of feature map being N×N×3. Next, a patch embedding layer is used to transform the feature map into sequence embedding, which consists of two steps: (1) the sinogram feature map is split into nonoverlapping patches with the size of n×n(n=4) and (2) these patches are projected a sequence with the number of $N^{2}/n^{2}$. After the patch embedding layer, the dimension of feature representation is $(N^{2}\times C)/16$. Following the patch embedding layer, the embedded patches pass through four encoder stages, which consist of the Swin transformer block and patch merging (only in the last three stages). The Swin transformer block uses window-based multihead self-attention (W-MSA) and shifted window-based multihead self-attention (SW-MSA) to compute the feature representations on the embedded patches. Each patch merging operation is used to 2× down-sample the embedding patches and expand to double the channels for the multiscale self-attention feature representations. Therefore, the output dimension of sequence feature representation for each encoder stage is $(N^{2}\times C)/16$, $(N^{2}\times 2C)/64$, $(N^{2}\times 4C)/256$, and $(N^{2}\times 8C)/1024$, respectively.

#### 2.3.2 Decoder: 2D convolutional

Because the resolution of the output image is higher than the resolution of feature maps from the locality module (introduced later), a generative CNN structure is utilized as the basic unit in the decoder stage. The decoder stage was built based on a 3×3 convolution with a stride of 1 followed by an up-sample layer. Specifically, after the last encoder stage, the feature map with a size of $\frac{N}{32}\times \frac{N}{32}\times 8C$ is up-sampled to $\frac{N}{16}\times \frac{N}{16}\times 8C$. At the following decoder stage, the feature maps are concatenated with the feature maps from the up-sample layer, and then the concatenated features are passed through the CNN block sequentially.

Through the whole decoder process, the size of feature map is up-sampled from $\frac{N}{32}\times \frac{N}{32}\times 8C$ to $\frac{N}{2}\times \frac{N}{2}\times C$. Instead of simple interpolation for up-sampling for the last output layer, a transposed convolution with a stride of 2 is applied. Finally, the image with the size of N×N×1 is obtained.

#### 2.3.3 Locality module as skip connection

To concatenate the encoder features from sinogram pixels and the features from the reconstructed image together for the decoder process, a locality module was first proposed instead of direct concatenation,^31^ as shown in Fig. 1(c). The sequence feature representation of an encoder stage is reshaped into 2D feature maps with $\frac{N}{4}\times \frac{N}{4}\times C$, $\frac{N}{8}\times \frac{N}{8}\times 2C$, $\frac{N}{16}\times \frac{N}{16}\times 4C$, and $\frac{N}{32}\times \frac{N}{32}\times 8C$, respectively. Then the locality module was added at the exit of each encoder stage, which was used to deliver the features to the decoder. As shown in Fig. 1(c), first, a CNN layer is used to capture local pixel information of the feature map. Next, the new feature map that passed through the CNN is reshaped into the flattened features, and a multilayer neural network (MNN) is used to learn the feature representation. Finally, the features are reshaped into a 2D feature map and passed into a CNN layer. Moreover, the residual learning mechanism is introduced to solve the overfitting problem in the deep learning network,^32^ and a skip connection is added between the input and output of the locality modules. The input and output of the locality module have the same feature size.

### 2.4 Datasets Preparation

**Fig. 2:**
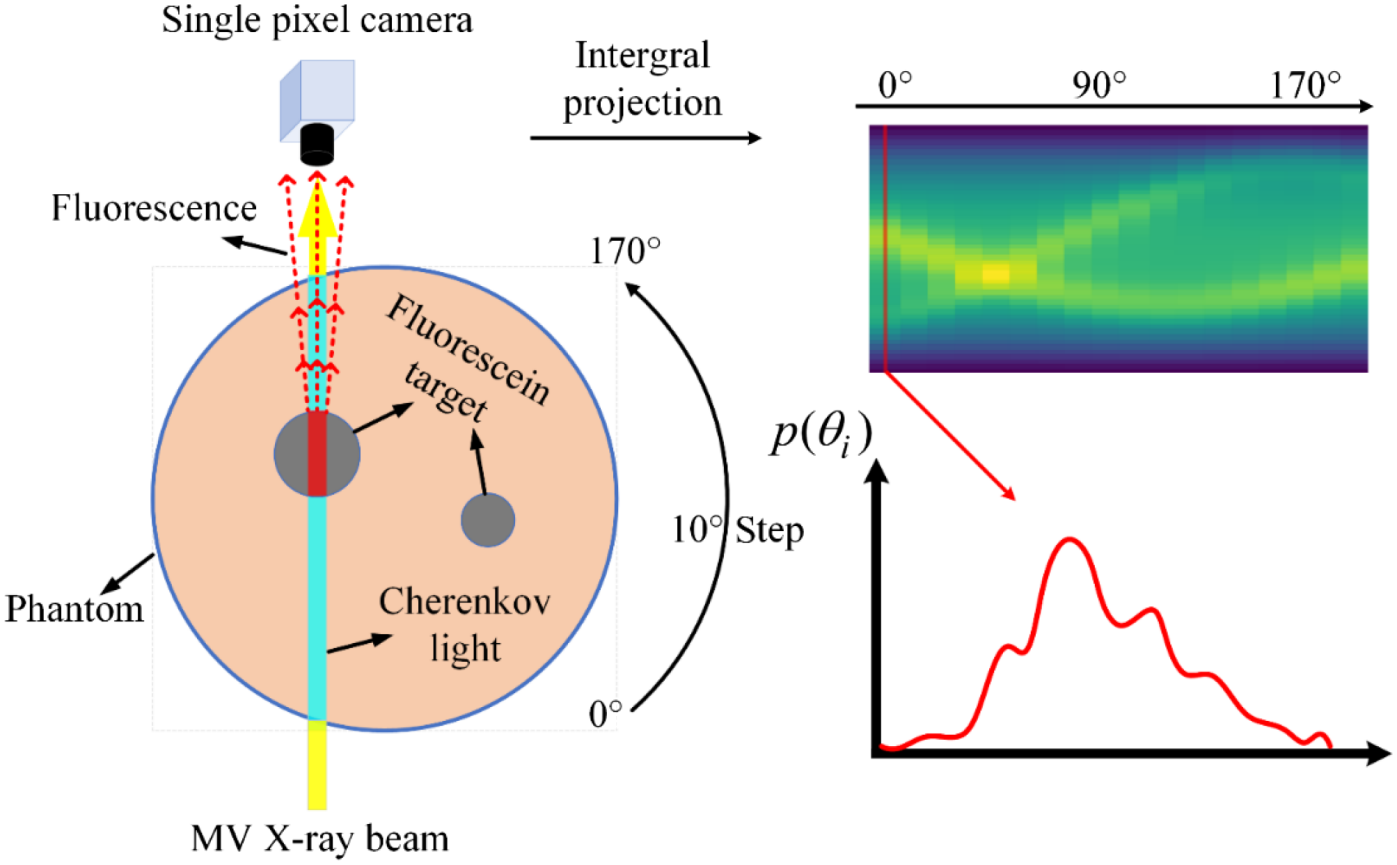
Schematic of generating sinogram data.

A circular phantom with a radius of 50 mm was used. The phantom was discretized into a mesh with 5133 finite-element nodes and 10,013 triangles elements. The optical properties used for simulation are shown in Table 1.^33^ Single or double fluorophores (contrast of 4:1 with background) with varied radii (from 4 to 8 mm) were placed at different positions inside the phantom. In total, 10,000 phantoms were obtained. The open-source software Nirfast was modified to generate sinogram data.^34^ 50 parallel beam sheets (step of 2 mm) were rotated from 0 deg to 170 deg in 10 deg intervals, and the emission signals were accumulated by integral detector along each beam source, as shown in Fig. 2. 1% random noise was added to the sinogram data. A bilinear interpolation was used to upscale the sinogram image from 18×50 to 128×128 to match the network input. Therefore, the size of the recovered fluorescein images was 128×128. The 10,000 datasets were divided into 8000 sets for training, 1000 sets for cross-validation, and 1000 sets for testing.

**Table 1:** Background optical properties of the phantom used to generate datasets.

Optical properties	$\mu_{ax}$	$\mu_{am}$	$\mu_{sx}$	$\mu_{sm}$	$\mu_{af}$
Unit (${\mathrm{mm}}^{-1}$)	0.009	0.006	1.314	1.273	0.008

### 2.5 Evaluation Metrics

To test the performance of the proposed reconstruction algorithm, three evaluation indicators were used.

Mean square error (MSE) is used to characterize the accuracy of reconstructed images and is defined as^35^

$$\mathrm{MSE}(\mathrm{GT},R)=\frac{1}{N^{2}}\overset{N}{\underset{i=1}{\sum }}\overset{N}{\underset{j=1}{\sum }}{\left(\mathrm{GT}(i,j)-R(i,j)\right)}^{2},$$ (7)

where GT and R are the ground truth and the reconstruction images with size of N×N, respectively.

Peak signal-to-noise ratio (PSNR) is used to measure image distortion or noise level between the ground truth and the reconstruction images and is defined as^36^

$$\mathrm{PSNR}(\mathrm{GT},R)=10\;\log_{10}[\frac{{\left(\mathrm{Max}(\mathrm{GT})\right)}^{2}}{\mathrm{MSE}(\mathrm{GT},R)}],$$ (8)

where Max(GT) is the maximum value of the ground truth.

Pearson correlation (PC) is used to measure the correlation between the ground truth and reconstructed images and is defined as^37^

$$\mathrm{PC}(\mathrm{GT},R)=\frac{\mathrm{cov}(\mathrm{GT},R)}{\sigma_{\mathrm{GT}}\sigma_{R}},$$ (9)

where $\sigma_{\mathrm{GT}}$ and $\sigma_{R}$ are the standard deviations of ground truth and reconstructed images, respectively and cov is the cross-covariance of ground truth or reconstructed images.

## 3 Experiments and Results

To demonstrate the performance of Swin-CNN, we compared it with the FBP and the AUTOMAP algorithms.

### 3.1 Numerical Phantom Experiments

#### 3.1.1 Single target experiment

**Fig. 3:**
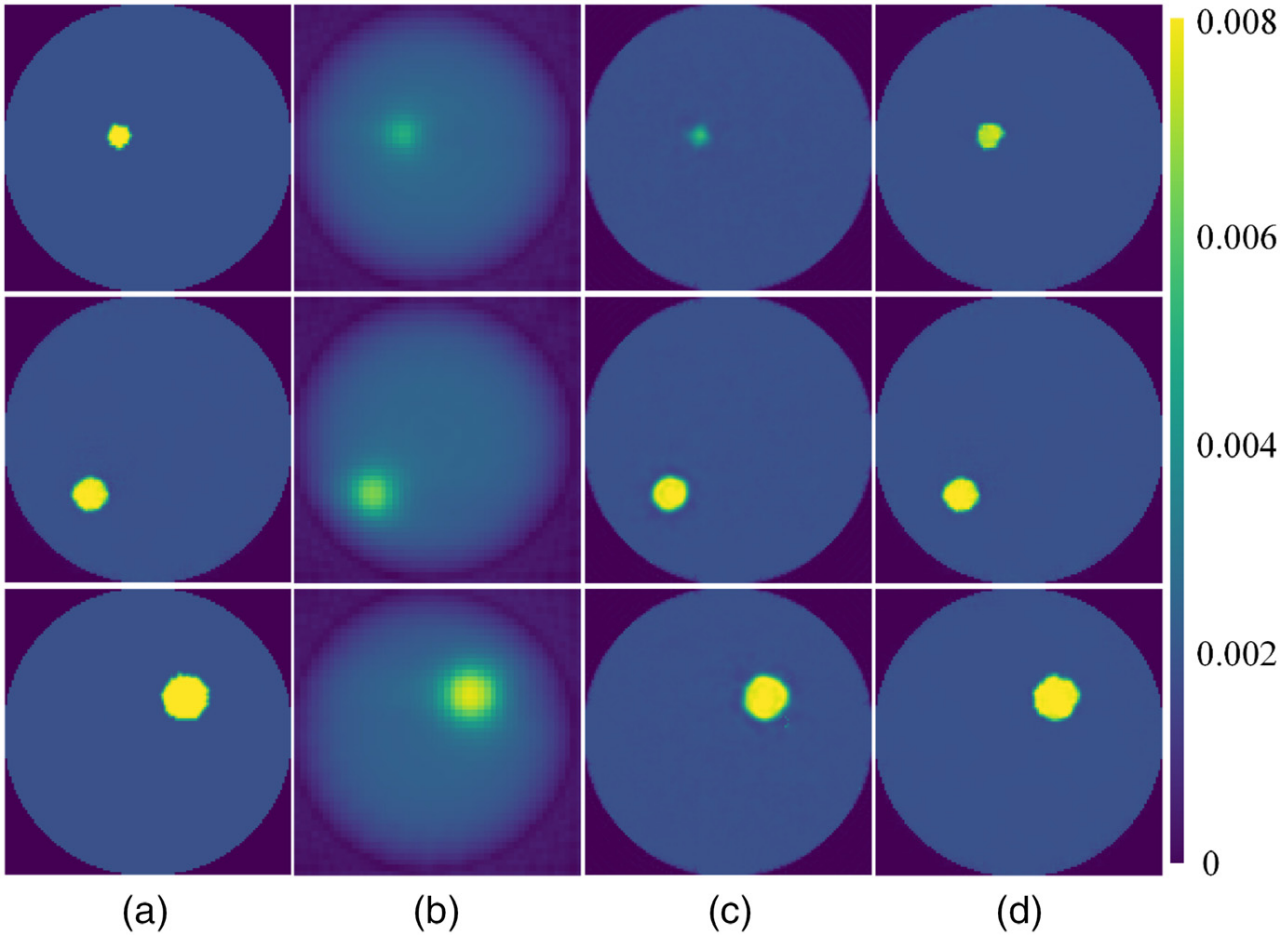
Reconstructed images for different algorithms. (a) The ground truth images, (b)–(d) the results reconstructed by FBP, AUTOMAP, and Swin-CNN, respectively. The radius of fluorescein target from the top to bottom rows are 4, 6, and 8 mm, respectively.

Figure 3 shows the reconstructed results in the case of single target. Figure 3(a) shows the ground truth with different radii and positions. Figures 3(b)–3(d) show the reconstructed images with the FBP, AUTOMAP, and Swin-CNN algorithms, respectively. From Fig. 3, we can observe that the FBP obtains the poorest reconstructed images with artifacts and blurred edges because only 18 projections were used. Compared with the FBP, AUTOMAP obtains better images, but there are still distortions and boundary artifacts in the reconstructed images. In contrast, the Swin-CNN algorithm obtains the best results with reduced artifacts and sharp edges.

**Fig. 4:**
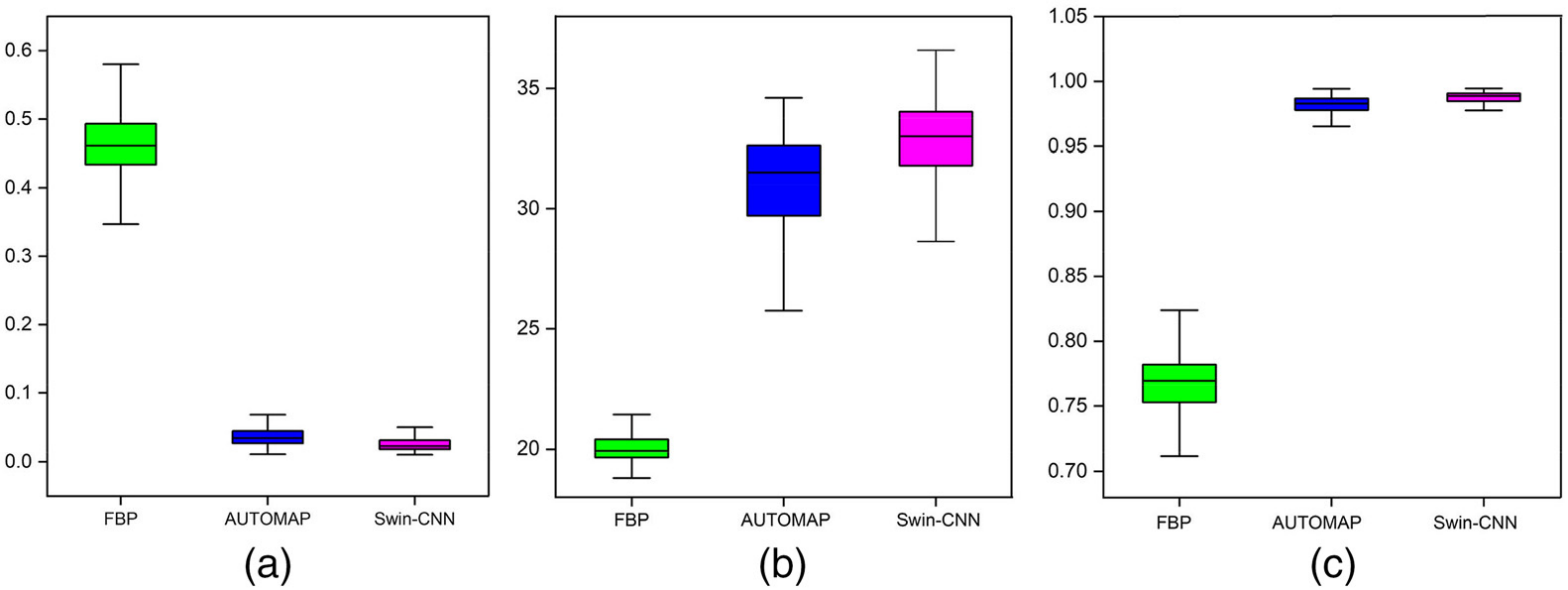
Statistical results for 1000 samples. (a) MSE, (b) PSNR, and (c) PC.

Table 2 shows the quantitative results for the three algorithms. Compared with the FBP method, PSNR and PC of the Swin-CNN are improved by >41.7% and 19.3%, respectively. Also, the Swin-CNN yields more than 5.7% and 1.1% improvements compared with the AUTOMAP method, respectively. Our results also demonstrate that the Swin-CNN obtains more quantitative accuracy and MSE is reduced by more than 94.1% and 33.3% over the FBP and AUTOMAP, respectively. Statistical results for 1000 samples are shown in Fig. 4. The results again demonstrate that the Swin-CNN yields superior performance compared with the other algorithms.

**Table 2:** Quantitative comparisons for the three algorithms in Fig. 3.

Radius	Method	MSE	PSNR (dB)	PC
4 mm	FBP	0.74	24.16	0.63
	AUTOMAP	0.08	30.57	0.95
	Swin-CNN	0.03	34.23	0.98
6 mm	FBP	0.47	22.86	0.76
	AUTOMAP	0.05	32.78	0.96
	Swin-CNN	0.02	35.08	0.99
8 mm	FBP	0.34	21.72	0.83
	AUTOMAP	0.03	33.22	0.98
	Swin-CNN	0.02	35.13	0.99

#### 3.1.2 Resolution experiment

**Fig. 5:**
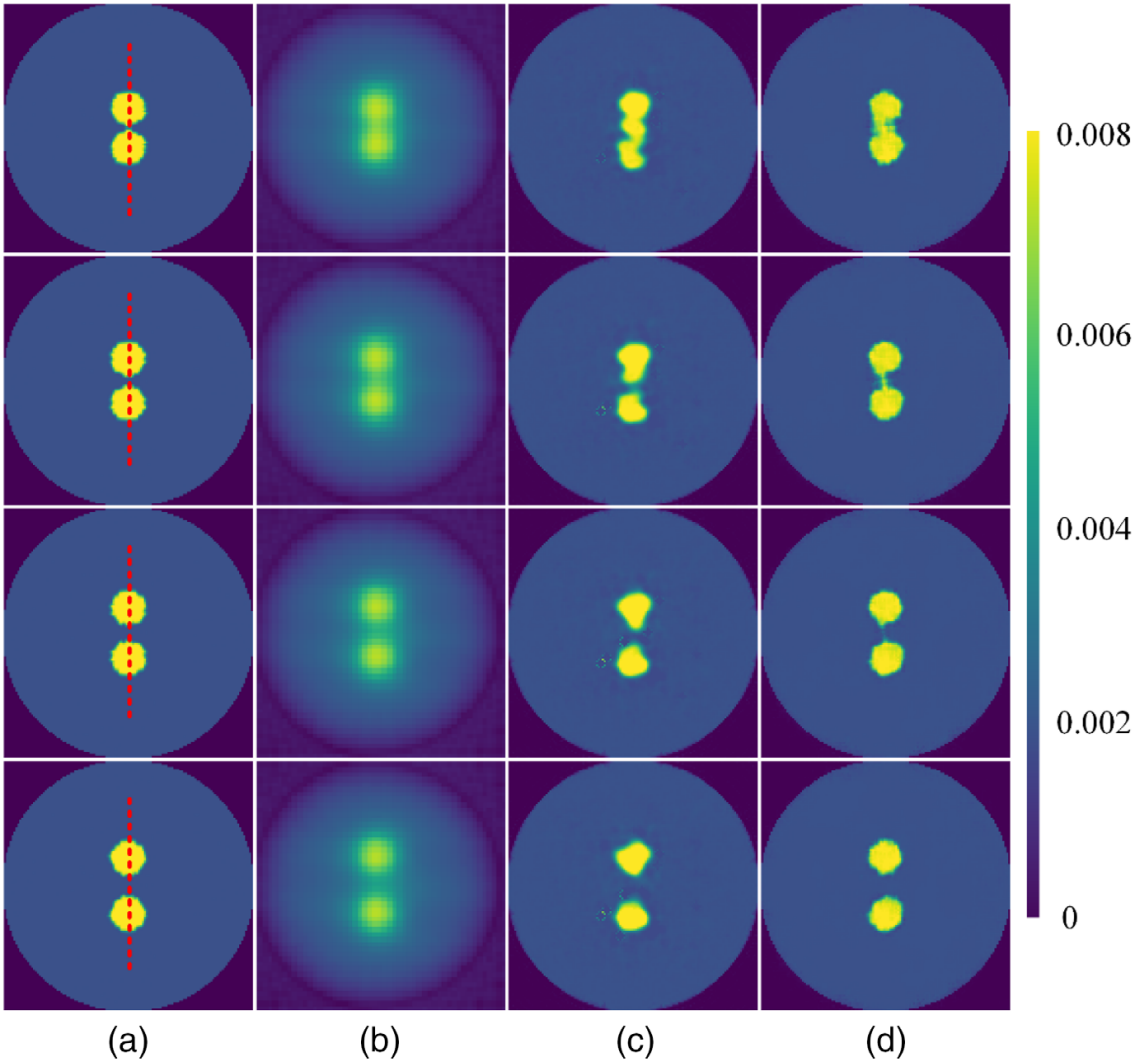
Reconstructed images for different algorithms. (a) The ground truth images, (b)–(d) the results reconstructed by FBP, AUTOMAP, and Swin-CNN, respectively. The edge-to-edge distance of two targets from the top to bottom rows is 2, 4, 6, and 8 mm, respectively.

We further test the ability of Swin-CNN to differentiate two targets. The edge-to-edge distance between two targets varied from 2 to 8 mm. The corresponding results are shown in Fig. 5. As shown in Fig. 5, the blurred images are again obtained for the FBP algorithm, and it is difficult to differentiate the targets when the edge-to-edge distance is 2 mm.

**Fig. 6:**
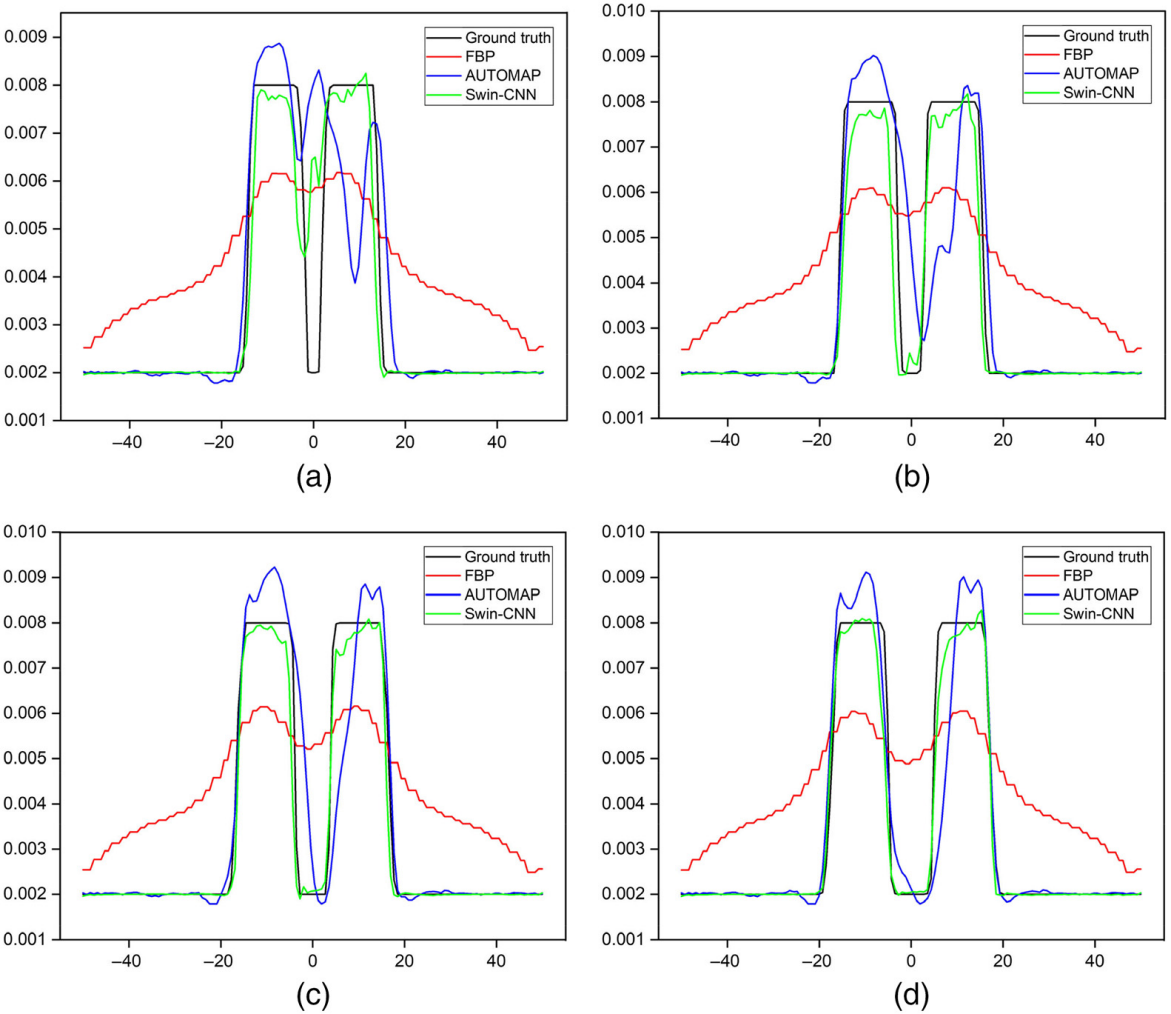
Profiles along the red dotted line in Fig. 5 with different edge-to-edge distances. (a) 2 mm, (b) 4 mm, (c) 6 mm, and (d) 8 mm.

Compared with the FBP, the AUTOMAP algorithm obtains much clearer images, but there are still artifacts and distortions around the targets. Figure 6 plots the profiles along the red dotted line shown in Fig. 5. The results again reveal that the values of the reconstructed images by the Swin-CNN are closer to their ground truth images. The quantitative comparisons for the three methods are compiled in Table 3. The results further demonstrate that the Swin-CNN obtains the best performance in terms of MSE, PSNR, and PC. For example, the PSNR is improved by more than 51.5%, and 4.9% compared with the FBP and the AUTOMAP algorithms, respectively.

**Table 3:** Quantitative comparisons for all methods in Fig. 6.

Radius	Method	MSE	PSNR (dB)	PC
2 mm	FBP	0.41	19.28	0.8
	AUTOMAP	0.12	27.44	0.9
	Swin-CNN	0.05	29.21	0.96
4 mm	FBP	0.43	18.94	0.78
	AUTOMAP	0.09	27.73	0.91
	Swin-CNN	0.04	30.51	0.97
6 mm	FBP	0.44	18.83	0.78
	AUTOMAP	0.1	28.56	0.92
	Swin-CNN	0.05	29.96	0.96
8 mm	FBP	0.43	18.85	0.79
	AUTOMAP	0.08	29.98	0.93
	Swin-CNN	0.03	32.35	0.98

#### 3.1.3 Robustness experiment

**Fig. 7:**
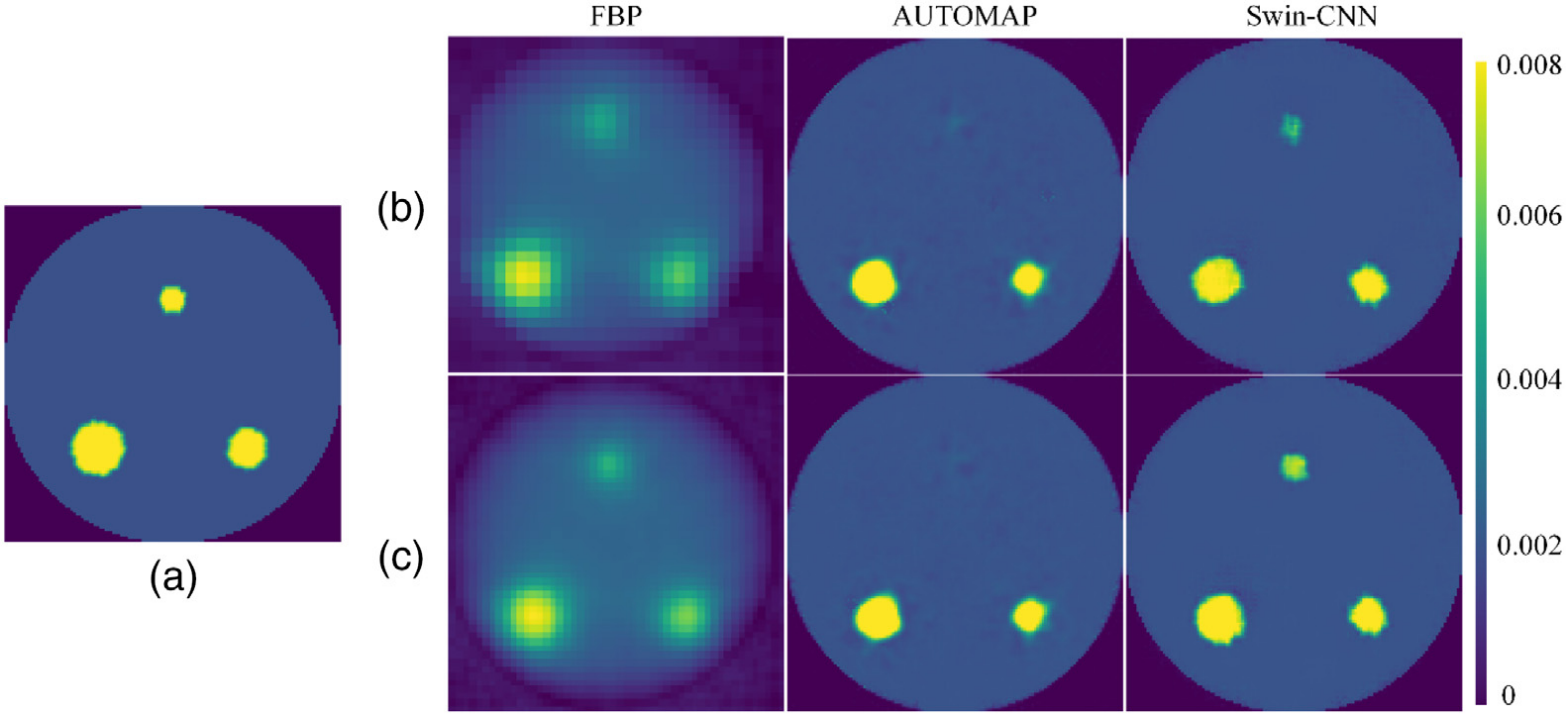
Reconstruction results with different numbers of parallel beams. (a) Ground truth image, (b) and (c) 30 and 50 parallel beams for each angular, respectively.

Furthermore, three targets with radii of 4, 6, and 8 mm were placed as shown in Fig. 7. 18 angular projections were measured with 10 deg intervals from 0 deg to 170 deg. Sinogram data was obtained for each angle with 30 parallel sheet scans or 50 parallel sheet scans. Figures 7(a) and 7(b) show the reconstruction results with 30 and 50 parallel sheet scans, respectively, and the quantitative results are compiled in Table 4. It can be observed that the performance of the three methods decreases with the decrease of scanning angles and sheet scans. The AUTOMAP algorithm fails to recover the distribution of the three targets because of incomplete data. In contrast, our results accurately recovers the distributions of the three targets. From Table 4, we can see that more parallel sheet scans contain better image quality and quantitative accuracy. For the Swin-CNN algorithm, the PSNR is improved from 16.28 dB (FBP) and 19.21 dB (AUTOMAP) to 25.37 dB when 30 scan beams were used.

**Table 4:** Quantitative comparisons for the three algorithms with 30 or 50 scan projections.

Number of scan beams	Method	MSE	PSNR (dB)	PC
30	FBP	0.68	16.28	0.66
	AUTOMAP	0.28	19.21	0.83
	Swin-CNN	0.21	25.37	0.87
50	FBP	0.41	18.45	0.79
	AUTOMAP	0.27	20.15	0.84
	Swin-CNN	0.11	29.07	0.94

### 3.2 Physical Phantom Experiments

**Fig. 8:**
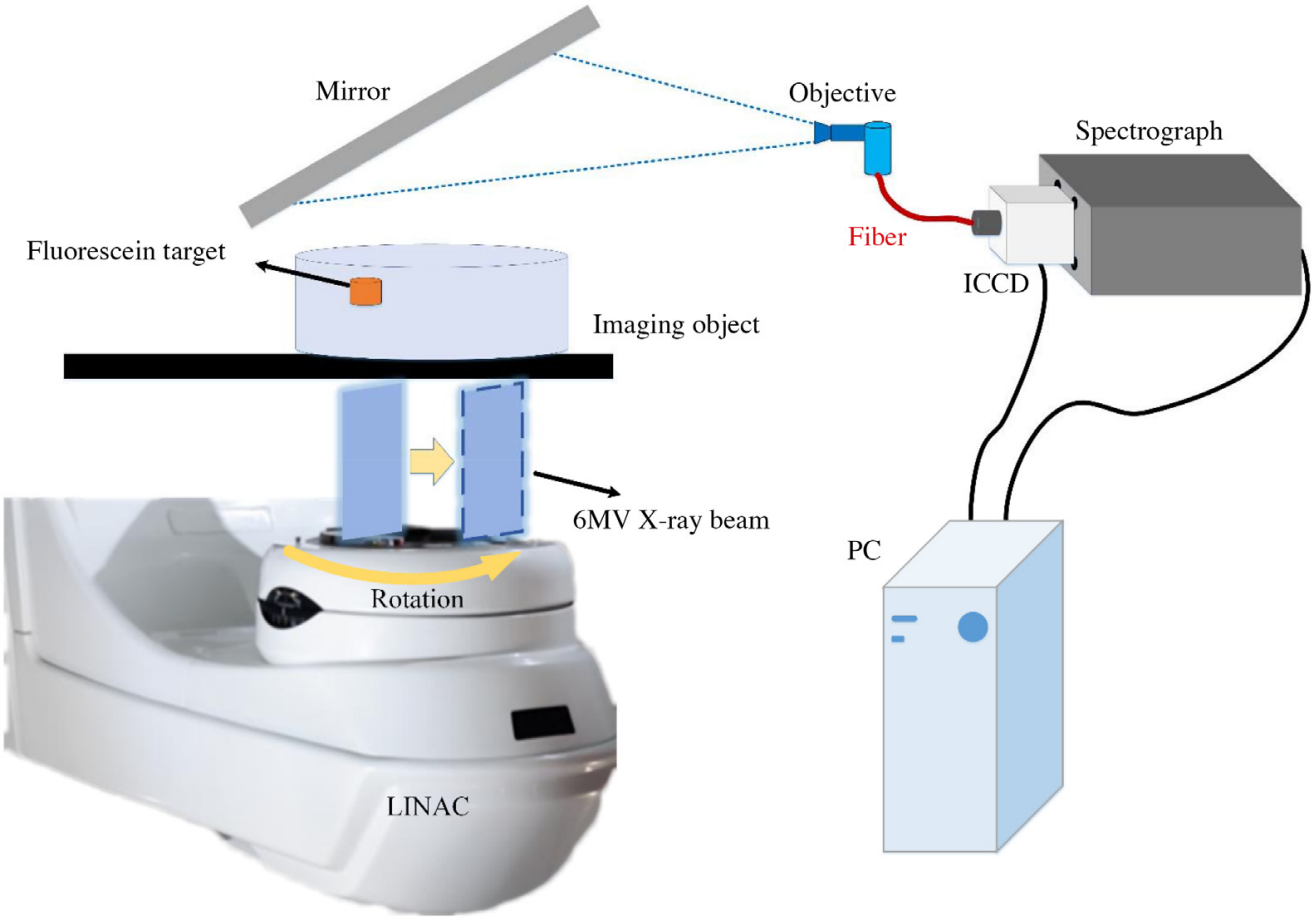
Schematic of data acquisition for physical phantom experiments.

To further evaluate the performance of Swin-CNN, physical phantom experiments were performed. Figure 8 shows the system used for data acquisition. Thin sheets of 6 MV x-ray beams were delivered from a clinical radiotherapy LINAC (Varian LINAC 2100CD, Varian Medical Systems, Palo Alto, CA) with a dose rate of 600 MU/min. A cylindrical water tank with a diameter of 100 mm and height of 80 mm was used as the imaging phantom. It was filled with 1% Intralipid (diluted from 10% Intralipid, Sigma–Aldrich) mixed with 1% porcine blood (Lamphire Inc., Pipersville, PA). The fluorescein target was a small plastic cylinder with an inner diameter of 10 mm and height of 10 mm filled with 500  μM fluorescein and located 1 mm below the liquid level in the cylinder tank. More details about the imaging system can be found in Ref. 6.

**Fig. 9:**
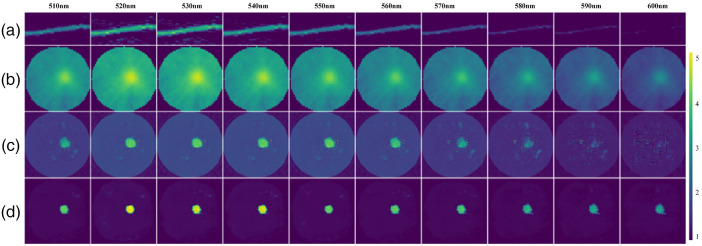
Physical phantom results with single fluorescein target. (a) Sinograms for fluorescence emission wavelength, (b)–(d) reconstructed images by FBP, AUTOMAP, and Swin-CNN, respectively.

**Fig. 10:**
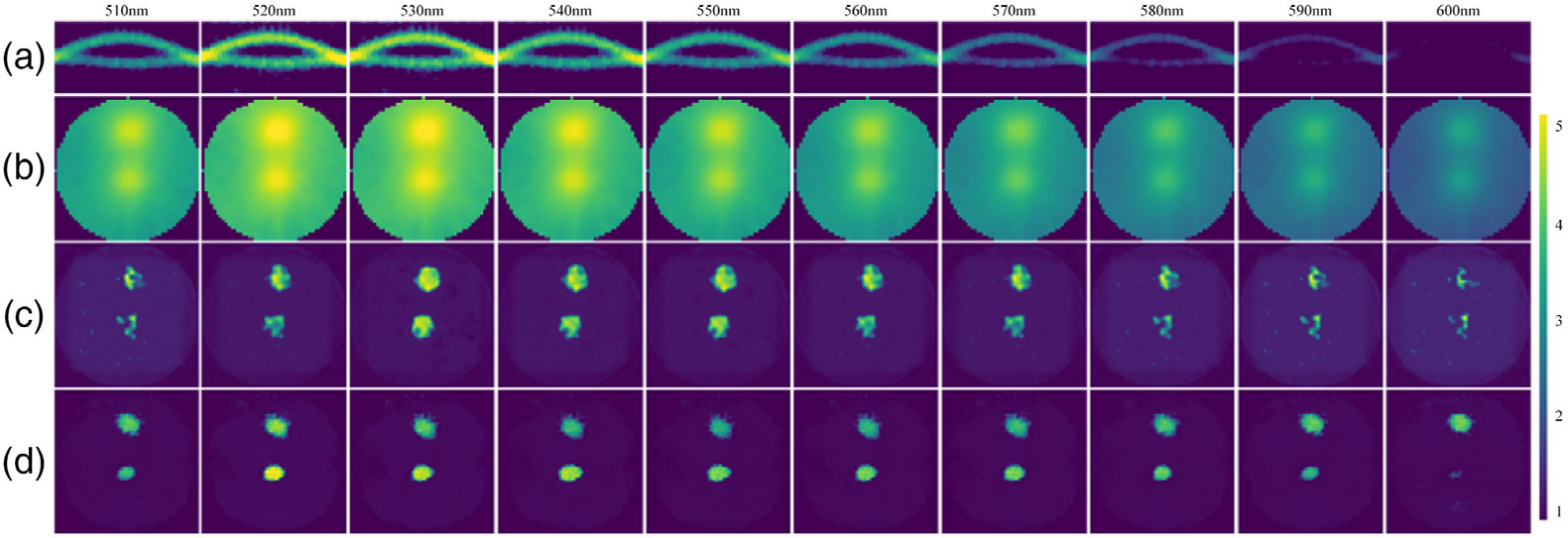
Physical phantom results with two fluorescein targets. (a) Sinograms for fluorescence emission wavelength, (b)–(d) reconstructed images by FBP, AUTOMAP, and Swin-CNN, respectively.

A total of 18 projections were obtained from 0 deg to 170 deg with angular steps of 10 deg, and 50 parallel sheet scans were horizontally translated by MLC with each step of 2 mm for each projection. For each scan, the spectrum of the whole optical signal of the imaging phantom could be measured ranging from 400 to 850 nm by the spectrometer. The sinogram of a single wavelength could be obtained through a linear spectral unmixing process.^6^^,^^38^ Figure 9(a) shows the sinograms for wavelengths of 510 to 600 nm, and (b)–(d) that are the reconstructed images by the FBP, AUTOMAP, and Swin-CNN, respectively. From Fig. 9(b), we can see that many artifacts exist in the reconstructed images by the FBP. In contrast, the AUTOMAP improves the reconstruction images and has much less artifacts and better signal localization. Among the three algorithms, Swin-CNN yields the best performances and obtains much clearer images. Figure 10 further shows the results with two fluorescent targets. Again, our algorithm obtains the better results. Note that the Swin-CNN was trained only with a single wavelength data, but it can be successfully used for other wavelengths. The results reveal that the Swin-CNN has low requirements on training data and good generalization properties.

### 3.3 *In Vivo* Experiments

**Fig. 11:**
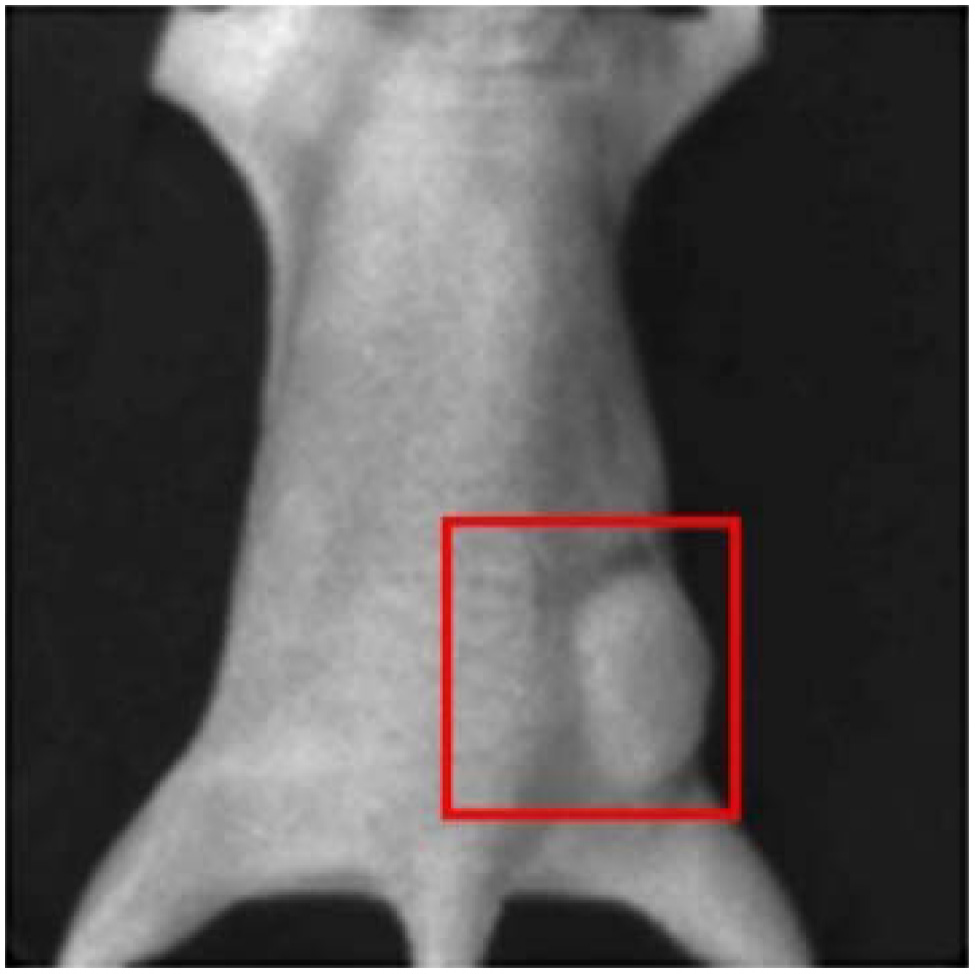
Fluorescent probe locally injected into the tumor (red box).

**Fig. 12:**
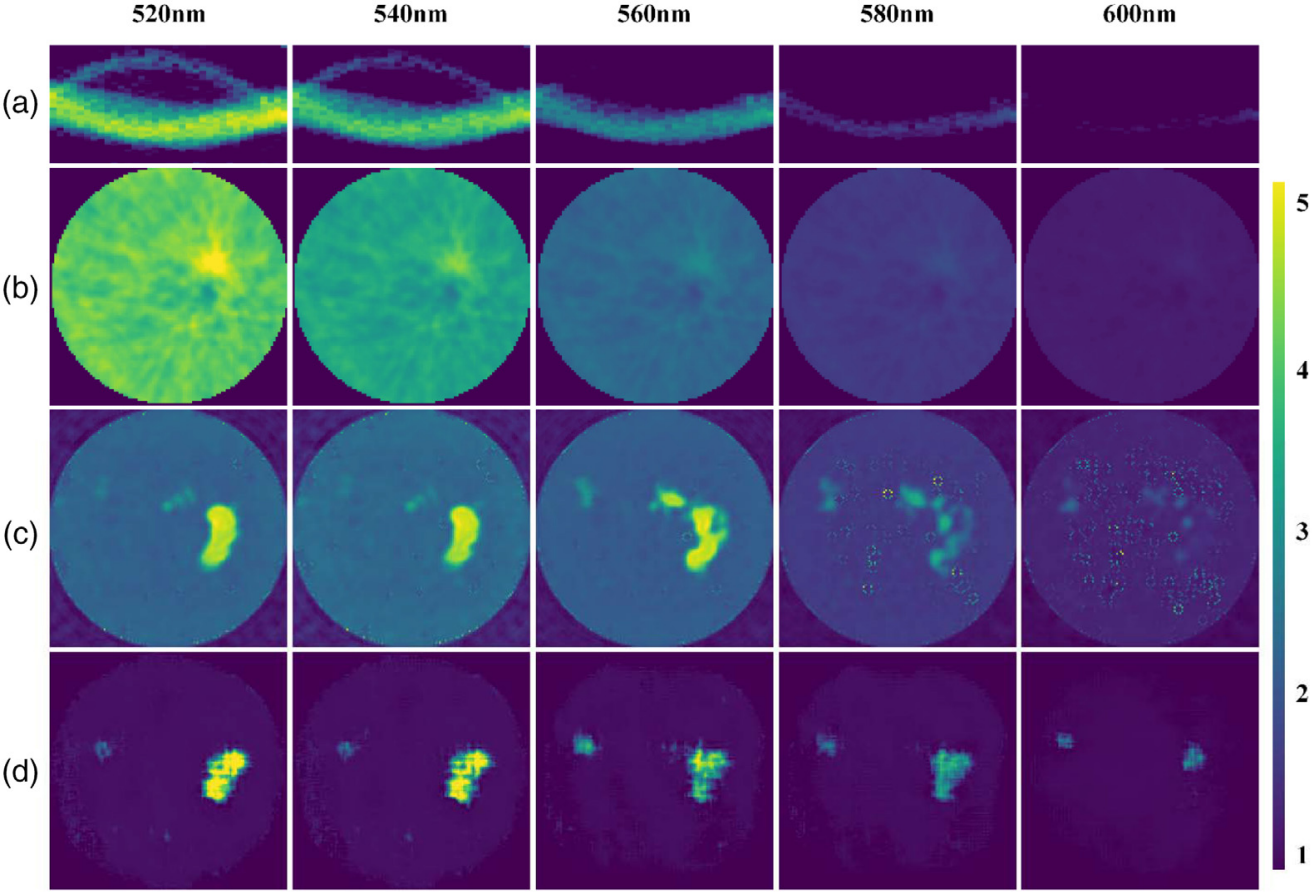
*In vivo* experimental results. (a) Sinograms for different fluorescence emission wavelength, (b)–(d) reconstructed images by FBP, AUTOMAP, and Swin-CNN for different wavelengths, respectively.

To further demonstrate the performance of Swin-CNN, an *in vivo* mouse experiment was performed. Experimental procedures involving live animals were carried out in accordance with the protocols approved by Dartmouth Institutional Animal Care and Use Committee (Protocol Numbers 00002173). The fluorescein was locally injected into the tumor on the flank of the mouse as shown as Fig. 11. The data acquisition was the same with the physical phantom experiment. Figure 12 shows the acquired sinogram data for five wavelengths and the corresponding reconstruction results. Our results demonstrate that the FBP method can only locate the tumor for the wavelength of 520 nm. In addition, there are significant artifacts in the background images. The AUTOMAP also fails to reconstruct the distribution of the tumor for the wavelength of 580 or 600 nm. In contrast, the Swin-CNN again obtains better images with less artifacts. The results also show that the Swin-CNN trained on simulation datasets can be directly extended to *in vivo* data, which reveals its good generalization properties.

## 4 Discussions

This work proposed and tested a deep learning-based reconstruction algorithm for XCLT that uses the unique approach to this problem of combining a Swin Transformer block and a CNN. We demonstrated that the transformer-based method can extract strong features from raw sinogram data and utilize the CNN to recover the image from the high-level feature representation. Meanwhile, the locality module was designed to learn the information of neighboring pixels in sinogram patches for connecting the encoder and the decoder. Numerical simulation, physical phantom, and *in vivo* experiments were used to test its performances.

The numerical experiments demonstrated that the Swin-CNN reconstruction significantly improved the quality of reconstructed images compared with the FBP and AUTOMAP approaches (Figs. 3 and 5). The FBP is preferred for its efficiency and fast calculation time; however, it results in artifacts from low contrast recovery and blurring, especially when the measured sinogram is insufficient. The AUTOMAP method requires training data for a near-optimal reconstruction mapping between the sinogram and the fluorescein images, which can reduce artifacts and blur in reconstructed images. However, because the overfitting of the fully-connected layers exists in AUTOMAP, the trained network based upon the training dataset with only single or double inclusions could not handle the three-inclusion case (Fig. 7). The Swin-CNN approach takes the sinogram reconstruction as a deep supervised learning task that uses the Swin Transformer to extract the information from the sinogram domain and the CNN to reconstruct the image through high-level feature representation.

The physical phantom experiments showed that the Swin-CNN reconstructs fluorescent targets at 510 to 600 nm wavelengths, which reveals nearly a full contrast recovery. Because the measured sinogram intensity was pretty weak at 600 nm and the information provided by the sinogram data was limited, the reconstructed image of Swin-CNN was poor compared with the FBP and AUTOMAP methods. For *in vivo* experiments, the proposed algorithm also shows good reconstruction results. However, to date, we only acquired very limited physical phantom and *in vivo* data; hence, we did not further test the performance of the Swin-CNN. Future work will be needed to collect more experimental data to test its performance. The performance of the Swin-CNN is affected by the inputted sinogram. It can be improved by increasing the parallel sheet scan number and signal-to-noise ratio during sinogram acquisition.

**Fig. 13:**
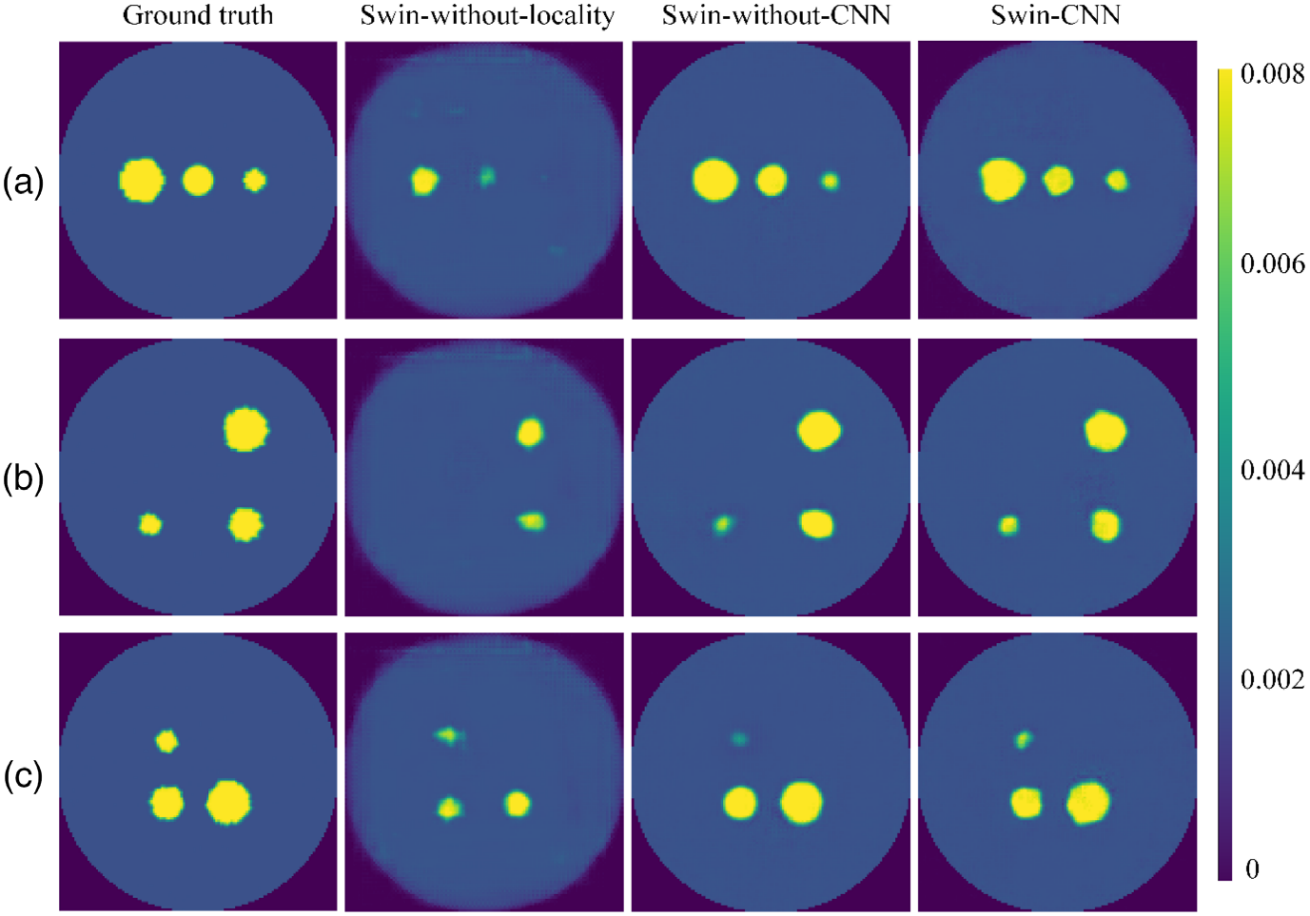
Reconstruction results for the variant of the proposed Swin-CNN. (a)–(c) The results when three fluorescent targets were placed at different positions.

**Fig. 14:**
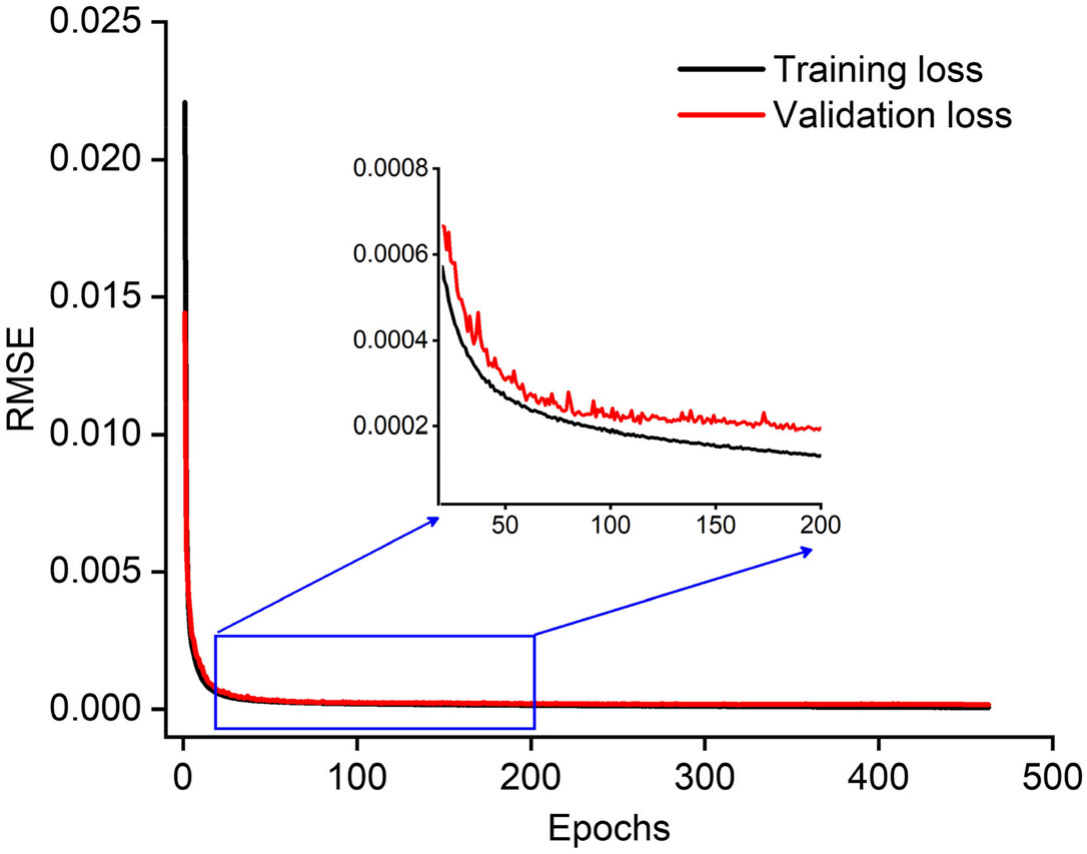
Performance plot of training and validation dataset for the Swin-CNN. Insets show a zoom-in on the marked blue region. Black and red lines represent the loss of the training and validation datasets, respectively.

For the ablation studies of the locality module, we analyzed this within the Swin-CNN. We only used concatenating connections without the locality module as the variant of Swin-CNN (Swin-without-locality). The reconstruction performance of Swin-CNN was also investigated when the two CNN layers of the locality module were replaced by concatenation operations (Swin-without-CNN). Figure 13 shows the results with three targets, and quantitative comparisons were reported in Table 5. The Swin-CNN achieves the best PC and the highest PSNR, which demonstrates the effectiveness of this module approach. Table 6 further shows that the Swin-CNN greatly reduced the training parameters compared with the AUTOMAP. The input of AUTOMAP contained fully connected layers, which makes the AUTOMAP scale linearly with the input size. However, the computing complexity (FLOPs) of the Swin-CNN is higher than the AUTOMAP, showing that the global self-attention of the image requires a lot of computation. Figure 14 illustrates the training and validation losses versus the number of epochs for the Swin-CNN. As can be seen, the validation loss curves closely follow corresponding training loss curves, showing the generalization ability of the Swin-CNN.

**Table 5:** Ablation studies of the locality module on Swin-CNN.

Method	MSE	PSNR (dB)	PC
Swin-without-locality	0.38 ± 0.03	17.07 ± 0.26	0.79 ± 0.02
Swin-without-CNN	0.09 ± 0.01	23.05 ± 0.5	0.92 ± 0.01
Swin-CNN	0.04 ± 0.01	26.7 ± 0.8	0.97 ± 0.01

**Table 6:** Number of training parameters and floating points of operations (flops) for the deep learning method.

Method	# Parameter	FLOPs
AUTOMAP	805.44 M	2.54 G
Swin-CNN	46.24 M	3.59 G

There have been many iterative reconstruction algorithms for optical tomography. For example, Cai et al.^39^ developed a non-negative iterative convex refinement approach for Cerenkov luminescence tomography (CLT). However, these iterative algorithms have to use the diffusion equation to model light propagation. After solving the diffusion equation with finite-element method, an optimization algorithm is essential for minimizing the difference between the calculated and measured surface optical fluxes. Therefore, iterative approaches are complex in terms of computations. Hence, we did not compare iterative reconstruction algorithms.

**Fig. 15:**
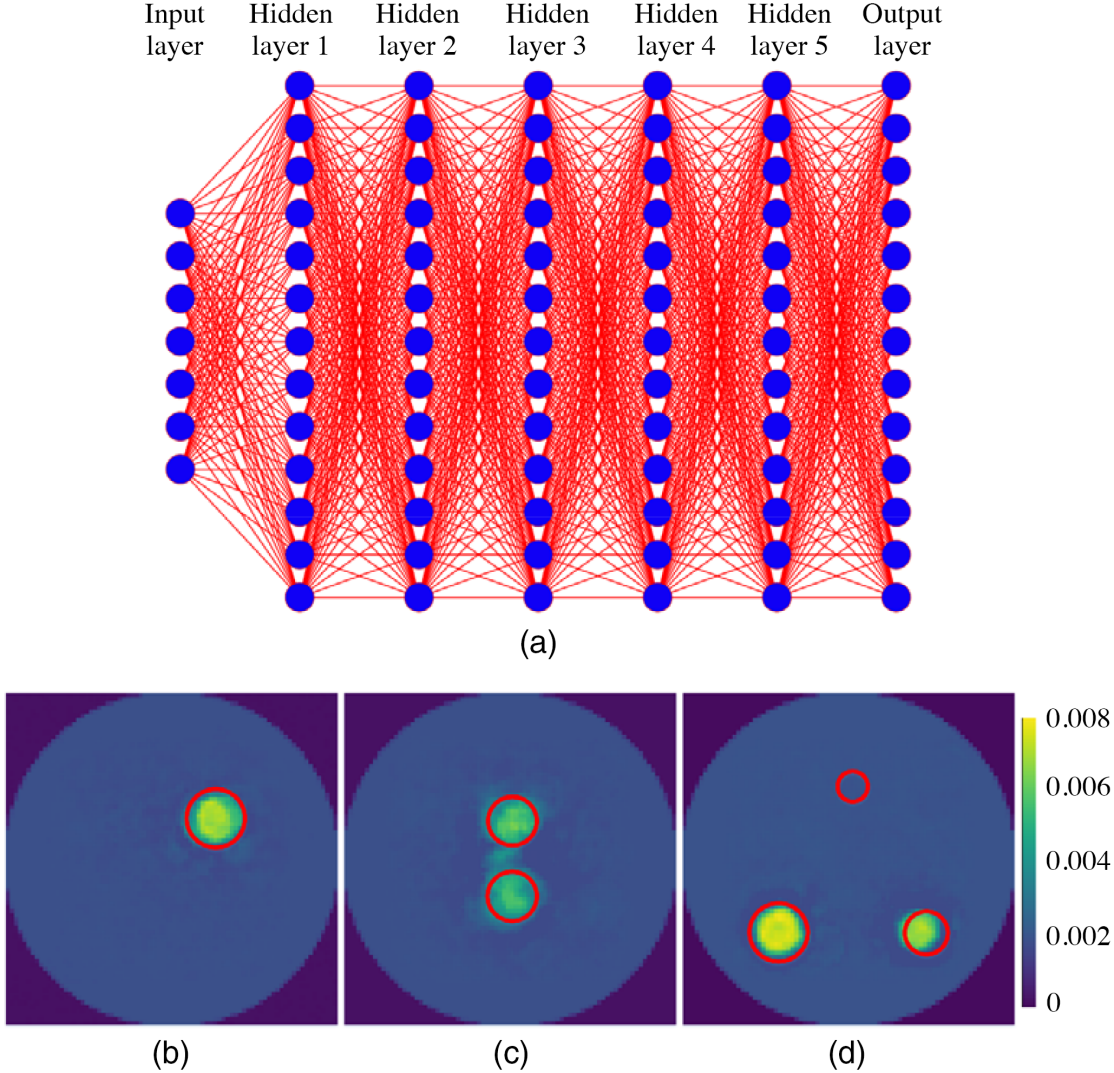
Results with the multilayer fully connected neural network (MFCNN). (a) The architecture of MFCNN, and (b)–(d) the reconstructed images with different numbers of targets. The red circles represent the real positions of targets.

Recently, there has been increasing interest in optical reconstruction based on multilayer fully connected neural network (MFCNN).^40^ We extended the MFCNN to XCLT and tested its performance with the single and multiple targets used in Figs. 3, 5, and 7. Its network architecture is similar to the architecture used by Zhang et al.,^40^ as shown in Fig. 15(a). More details about the MFCNN can be found in Ref. 40. Its input was the raw sinogram image, and the output was the reconstructed XCLT image. The corresponding results are shown in Figs. 15(b)–15(d). Compared with Figs. 3, 5, and 7, we can see that there are significant artifacts in reconstructed images by the MFCNN, whereas the Swin-CNN obtains clean images. Also, the MFCNN fails to reconstruct the target with a small size. Our algorithm produces significantly better results over the MFCNN. The reason is that the Swin-CNN can extract more global and local features from sinograms. We also observed that the number of neurons in hidden layers should not be changed once the MFCNN is trained well. Therefore, when imaging an object that is different from one used for training, the generalization performance of the MFCNN is limited.

## 5 Conclusion

Here, we proposed a deep learning algorithm with three distinct components as a reconstruction algorithm for CELST. This included a (1) transformer to encode information, (2) a CNN to reconstruct the images, and (3) a locality module to link the encoder to the decoder. A major success was that, even though the network was trained with numerical phantoms datasets, the trained network was able to directly reconstruct images from physical phantom data and *in vivo* mouse data. The proposed Swin-CNN inherited the merits of both a Swin transformer for feature extraction and a CNN for image reconstruction. Moreover, the locality module introduced into the encoder and decoder was able to learn features between adjacent pixels on the sinogram image data. Future work will include incorporating real data into the network training to improve the performance and further refine its performance in more complex tissue samples.
